# Best evidence summary for the management of epidermal growth factor receptor inhibitor-induced skin toxicity symptoms in cancer patients

**DOI:** 10.3389/fmed.2025.1665823

**Published:** 2025-11-25

**Authors:** Kexuan Li, Xiuyue Qiu, Jiayu Zhang, Yingshuyi Zhou, Youfen Fan, Qionghui Sun

**Affiliations:** 1 Burn Department, Ningbo No.2 Hospital, Ningbo, Zhejiang, China; 2 School of Nursing, Zhejiang Chinese Medical University, Hangzhou, Zhejiang, China; 3 School of Nursing, Zhejiang Changzheng Vocational & Technical College, Hangzhou, Zhejiang, China; 4 Department of Gastrointestinal Surgery, Ningbo No.2 Hospital, Ningbo, Zhejiang, China

**Keywords:** cancer, targeted therapy, epidermal growth factor receptor inhibitors, skin nursing, summary of evidence

## Abstract

**Objective:**

This study aimed to evaluate and summarize the best available evidence on the management of epidermal growth factor receptor inhibitor-induced skin toxicity symptoms in cancer patients, serving as a reference for medical staff.

**Methods:**

We systematically searched for evidence on skin toxicity symptoms in databases such as China National Knowledge Infrastructure (CNKI) and Wanfang; guideline repositories such as Guidelines International Network (GIN) and New Zealand Guidelines Group (NZGG); and professional organization websites such as International Union Against Cancer (UICC) and American Cancer Society (ACS). The search time was limited from database establishment to July 2024. Two researchers evaluated the quality of the literature and extracted the data.

**Results:**

A total of 20 articles were included in this study, including 3 clinical decisions, 7 guidelines, 3 evidence summaries, 2 recommended practices, and 5 expert consensuses. Finally, 27 pieces of evidence were identified across 9 aspects: professional medical training, patient health education, precise skin assessment, reducing skin irritation, promoting skin comfort, skin sun protection care, skin moisturizing care, drug preventive measures, and drug treatment measures.

**Conclusion:**

Our research summarizes the best evidence for the management of epidermal growth factor receptor inhibitors (EGFRI)-induced skin toxicity symptoms in cancer patients. In clinical practice, it is necessary to fully consider the clinical situation, balance professional judgment with patients’ preferences, follow the principle of individualization, analyze the obstacles and facilitating factors of the application of evidence, and apply the evidence to clinical practice prudently.

## 1 Introduction

Epidermal growth factor receptor inhibitors (EGFRIs) stand out as the most frequently used class of targeted agents and are widely used in the management of diverse malignancies, including lung, pancreatic, head and neck, and colorectal cancer (1). EGFRIs function by inhibiting specific molecular pathways that regulate tumor cell proliferation, thereby enhancing clinical response rates and extending the overall survival of individuals with cancer (2). Although EGFRIs can antagonize the progression of cancer, they can also damage the skin and its appendages. Cutaneous toxicity represents the most prevalent side effect associated with EGFRIs (3), typically manifesting as a spectrum of symptoms such as acneiform rash, xerosis, fissuring, exfoliation, and pruritus (4), with a staggering overall incidence rate of up to 90% (5). The impact of these dermatologic toxicities extends beyond the mere physical, affecting patients’ psychological wellbeing and overall quality of life (6). Patients who struggle to endure these symptoms may experience treatment interruptions, dosage adjustments, or even discontinuation of therapy (7). Given the significance of these issues, the management of EGFRI-induced cutaneous toxicities is paramount to optimize therapeutic outcomes. Therefore, this study aims to evaluate and summarize the best available evidence for managing EGFRI-induced skin toxicity, with the goal of providing a reference for medical staff.

## 2 Materials and methods

### 2.1 Retrieval strategy

Researchers conducted evidence retrieval from top to bottom in accordance with the “6S” evidence model. They adopted a combination of subject headings and free words, using “cancer/tumor” “target/epidermal growth factor/EGF” “side effects/adverse reactions/adverse events/toxicity/skin lesions/rash/drug eruption” as search terms in Chinese, and “tumor/cancer/neoplasm/oncology/carcinoma,” “target/epidermal growth factor/EGF,” “side effects/adverse reactions/adverse events/toxicity/skin/dermatology/exanthema/rash/acneiform eruption” as search terms in English. The databases included UpToDate, British Medical Journal (BMJ), Zynx, DynaMed, Cochrane Library, Joanna Briggs Institute Library Evidence-Based Health Care Library (JBI), Cumulative Index to Nursing and Allied Health Literature (CINAHL), Web of Science (WoS), PubMed, Embase, Proquest, Chinese Biomedical Database (CBM), China National Knowledge Infrastructure (CNKI), Wanfang, Chinese Scientific Journal Database (VIP), etc. The guidelines websites included the World Health Organization (WHO) website, Medlive, American Society of Clinical Oncology (ASCO), Scottish Intercollegiate Guidelines Network (SIGN), Institute for Clinical and Economic Review (ICER), the New Zealand Guidelines Group (NZGG), National Institute for Health and Care Excellence (NICE), Guidelines International Network (GIN), National Guideline Clearinghouse (NGC), etc. The professional society websites included European Society of Medical Oncology (ESMO), National Cancer Institute (NCI), American College of Physicians (ACP), UICC, American Cancer Society (ACS), Registered Nurses Association of Ontario (RNAO), International Council of Nurses, etc. The search was conducted with no start date restriction, and the end date was set to July 2024 to include all available evidence from the inception of each database.

### 2.2 Inclusion and exclusion criteria

The inclusion criteria for literature were as follows: The research subjects are adult cancer patients using EGFRIs drugs; the research content involves the assessment, prevention, and management of skin toxicity symptoms; the outcome indicators include the incidence of skin toxicity symptoms, the degree of improvement in patients’ subjective experience, the awareness rate of skin toxicity, treatment compliance, quality of life, etc.; the research types are clinical decisions, guidelines, evidence summaries, recommended practices, and expert consensuses.

The exclusion criteria for literature were as follows: Literature that was a duplicate publication, incomplete in information, or unable to obtain the full text; related literature such as conference papers and news reports; literature with methodological flaws or low quality as determined by our standardized appraisal process; and non-Chinese and non-English literature.

### 2.3 Literature screening process

Two members of the research team independently screened and extracted the literature. In case of disagreements, they would conduct analysis and discussion or consult a third researcher for assistance in judgment. First, the EndNote software was used for literature screening and duplication removal. Second, the titles and abstracts were read to exclude obviously irrelevant literature. Finally, the full texts were carefully read to determine the ultimately included literature.

### 2.4 Literature quality evaluation

Two members of the research team independently conducted the quality assessment of the included literature. Different methodological quality assessment criteria for literature were selected based on the types of research. Guidelines were evaluated using the Appraisal of Guidelines for Research and Evaluation II (AGREE II) (8); expert consensuses were evaluated using the evaluation criteria for expert consensuses of the Joanna Briggs Institute (JBI) Evidence-based Health Care Center in Australia (9). For evidence summaries, recommended practices, etc., the quality assessment was carried out by tracing the references and based on the original literature corresponding to the extracted evidence items. In cases where it was difficult to determine whether a literature should be included in this study or when there were conflicting evaluation opinions, the decision was made by the third member.

## 3 Results

### 3.1 General characteristics of the included literature

**Figure 1:**
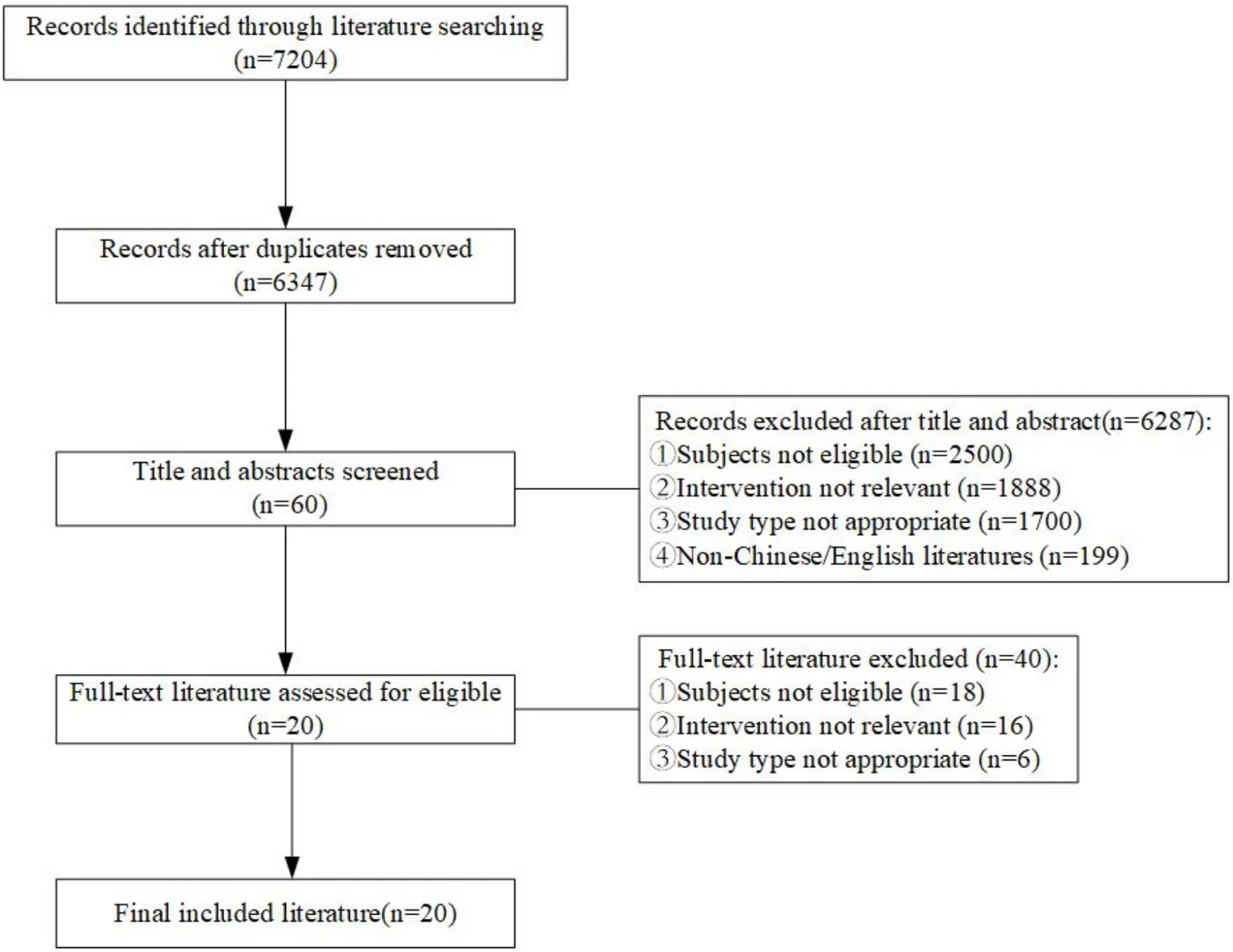
Flow diagram of the literature search and selection process.

A total of 7,204 literature were retrieved in this study. After duplicate removal, 6,347 remained. Following the initial screening, 60 literature were obtained. After full-text reading and further screening, 20 literature were selected, including 3 clinical decisions (10–12), 7 guidelines (13–19), 3 evidence summaries (20–22), 2 recommended practices (23, 24), and 5 expert consensuses (25–29). Analysis of the incorporated literature reveals that the core focus resides in pharmacological characteristics rather than cancer type-specific classification. Given that the pathogenesis of epidermal growth factor receptor inhibitor-induced cutaneous toxicity—primarily mediated through inhibition of the epidermal growth factor receptor (EGFR) signaling pathway in keratinocytes and follicular epithelium—exhibits remarkable consistency, corresponding management strategies demonstrate broad generalizability and translatability across various malignancies (e.g., non-small cell lung cancer, colorectal cancer, and head and neck cancer). Accordingly, this review emphasizes the synthesis of drug class-based, broadly applicable high-level evidence and management recommendations, with the goal of providing a unified and comprehensive reference for clinicians and patients undergoing EGFRIs therapy. The flow diagram of the literature search and selection process is illustrated in Figure 1. The general characteristics of the included literature are shown in Table 1.

**Table 1:** Characteristics of included literature (*n* = 20).

Author	Evidence type	Time	Literature theme	Source
Peng et al. (10)	Clinical decisions	2022	Skin adverse events with other biologic agents in molecularly targeted therapies and cancer therapies	UpToDate
Qu et al. (11)	Clinical decisions	2022	Acneiform rash secondary to EGFRIs and MEK inhibitors	UpToDate
Anthony et al. (12)	Clinical decisions	2022	Acneiform rash due to EGFRIs	Dynamed
Zhang et al. (13)	Guidelines	2019	Practice Guidelines for the Management of Cancer Skin Reaction Symptoms in China	CNKI
Cury-Martins et al. (14)	Guidelines	2020	Brazilian guidelines for the management of cutaneous adverse events caused by cancer therapy	Medlive
ACS (15)	Guidelines	2020	Management of cancer therapy-related skin side effects	ACS
Williams et al. (16)	Guidelines	2020	Guidelines on cancer-related skin toxicity	CINAHL
Lacouture et al. (17)	Guidelines	2021	Anti-cancer drug-related skin toxicity prevention and management	ESMO
Lacouture et al. (18)	Guidelines	2011	MASCC clinical practice guidelines for the prevention and treatment of EGFRI-associated skin toxicities	Pubmed
Califano et al. (19)	Guidelines	2015	UK EGFR tyrosine kinase inhibitor adverse event management	CINAHL
Hu et al. (20)	Evidence summaries	2022	Evidence summary for prevention and management of cutaneous adverse reactions in tumor-targeted patients	CNKI
Leaderlou and Magtoto (21)	Evidence summaries	2021	EGFRIs dermatologic toxicity management	JBI
Oerlemans (22)	Evidence summaries	2022	Prophylaxis and treatment of EGFRI-associated rash	JBI
Guo et al. (23)	Recommended practices	2018	Best evidence application for management of skin toxicities caused by EGFRIs	Wanfang
Kelly et al. (24)	Recommended practices	2020	Evidence-based nursing practice for EGFRIs associated skin toxicity	CINAHL
Melosky et al. (25)	Expert consensuses	2009	Canadian consensus on the management of skin rash during EGFR-directed monoclonal antibody therapy	Web of Science (WoS)
Potthoff et al. (26)	Expert consensuses	2011	German consensus on the interdisciplinary management of EGFRI-induced skin reactions	PubMed
Chu et al. (27)	Expert consensuses	2017	China–Taiwan consensus on prevention and management of EGFR–TKI-associated skin toxicities	PubMed
Hu et al. (28)	Expert consensuses	2019	Expert consensus on management of adverse reactions to EGFR–TKIs	CNKI
Wang et al. (29)	Expert consensuses	2021	Expert consensus on clinical management of anti-EGFR monoclonal antibody treatment-related cutaneous adverse reactions	CNKI

### 3.2 Quality evaluation results of the included literature

A total of 7 guidelines (13–19) were included in this study. The standardized percentages of each dimension of the guidelines and the results of two comprehensive evaluations are detailed in Table 2. Currently, there is no single tool to evaluate the literature quality of evidence summaries and recommended practices (9). Therefore, in this study, the method of tracing references was adopted for quality assessment. It was found that the overall quality of evidence summaries by scholars such as Hu et al. (20), Leaderlou and Magtoto (21), and Oerlemans (22) was relatively good, and they were approved for inclusion. Additionally, it was found that the quality of recommended practices by scholars such as Guo et al. (23) and Kelly et al. (24) was relatively good, and they were also approved for inclusion. A total of 5 expert consensus documents (25–29) were included in this study. The evaluation results of all items were “yes.” The research design was complete, and the overall quality was high; thus, they were approved for inclusion.

**Table 2:** Standardized scores and evaluation results in all areas of the guidelines.

Included guidelines	Percentage of standardization in each domain of the guide (%)						≥60% number of fields (number)	≥30% number of fields (number)	Recommended level
	Scope and purpose	Stakeholder involvement	Rigor of development	Clarity of presentation	Clarity of presentation	Editorial independence			
Zhang et al. (13)	94.44	88.89	96.88	100.00	83.30	75.00	6	6	A
Cury-Martins et al. (14)	72.22	80.56	40.63	91.97	66.67	95.83	5	6	B
ACS (15)	75.00	36.11	4.17	41.67	35.00	4.17	1	4	B
Williams et al. (16)	94.44	97.22	80.21	83.33	75.00	91.67	6	6	A
Lacouture et al. (17)	83.33	77.78	50.00	70.83	58.33	50.00	3	6	B
Lacouture et al. (18)	91.67	77.78	52.08	66.67	50.00	50.00	3	6	B
Califano et al. (19)	88.89	66.67	60.42	95.83	60.00	75.00	6	6	A

### 3.3 Evidence description and summary

In this study, evidence was summarized across 9 aspects: professional medical training, patient health education, precise skin assessment, reducing skin irritation, promoting skin comfort, skin sun protection care, skin moisturizing care, drug preventive measures, and drug treatment measures. A total of 27 pieces of evidence were formed, as detailed in Table 3.

**Table 3:** Best evidence summary for management of epidermal growth factor receptor inhibitor-induced skin toxicity symptoms in cancer patients.

Aspects	Evidence item	Evidence item	Recommendation level
Professional medical training	1. Through standardized training, medical staff systematically study skin care-related guidelines, expert consensus, etc., and master the symptoms, predilection sites, development process, severity grading, treatment principles, and precautions for commonly used drugs of EGFRI-induced cutaneous toxicity (12, 18, 23, 27).	5	A
	2. Establish a referral/consultation model for dermatology (29), and form a professional cutaneous toxicity team consisting of (oncologists, dermatologists, pharmaceutical experts, nursing teams, etc.) to implement interdisciplinary comprehensive management of moderate to severe cutaneous toxicity (14, 16).	5	A
Patient health education	3. Medical staff are recommended to inform cancer patients that acneiform rash is not a contraindication to the continued use of EGFRIs. There is a consistent positive correlation between the severity of acneiform rash and anti-tumor activity (10, 22), and it may be a surrogate marker for the therapeutic effect of EGFRIs (12, 26).	2	A
	4. The rash is non-infectious (28), self-limiting, and reversible (26). It follows a pattern of repeated recurrence-remission with the treatment cycle, and its severity generally decreases gradually (29). Typically, it subsides within 4 weeks after discontinuing EGFRIs and does not leave scars (11, 25).	3	A
	5. It is recommended that medical staff inform cancer patients and their families of the clinical manifestations, potential impacts and concerns, treatment effectiveness, and prognosis of cutaneous toxicity symptoms caused by EGFRIs (20, 23). Urge patients to report in a timely manner when relevant symptoms appear, and provide relevant health education and information resources to improve patients’ knowledge of EGFRIs and their self-skin-care levels (20, 22).	1	A
Precise skin assessment	6. Risk factors associated with cutaneous toxicity symptoms include drug-related factors (such as EGFRIs type, dosage, treatment duration, etc.) and patient-specific factors (such as gender, smoking status, skin phototype, immune status, history of receiving cytotoxic drugs, etc.) (10, 12, 29). Ultraviolet exposure of the skin, insufficient moisturization, and concurrent radiotherapy can exacerbate cutaneous toxicity symptoms in cancer patients (11, 20).	3	A
	7. It is recommended that medical staff evaluate the location, nature, severity, density, development process, triggering factors, presence of infection, patients’ subjective experiences, and reports and records of other adverse reactions of cutaneous toxicity symptoms caused by EGFRIs (17, 20).	5	A
	8. It is recommended to use skin assessment tools that are easily acceptable to patients, such as Common Terminology Criteria for Adverse Events (CTCAE), Multinational Association of Supportive Care in Cancer (MASCC) Skin Toxicity Scale (MESTT), Epidermal Growth Factor Receptor Inhibitor Related Skin Toxicity Index (EGFRISTI), Dermatology Life Quality Index (DLQI), Skindex, Functional Assessment of Cancer Therapy-Epidermal Growth Factor Receptor Inhibitor-18 (FACT-EGFRIs-18) Scale, etc. (14, 18, 20, 26), to assist in determining the grade of cutaneous toxicity (20).	5	A
	9. Medical staff should conduct follow-up evaluations of cancer patients taking EGFRIs at least once every 2 weeks to promptly identify and intervene in cutaneous toxicity symptoms (26) If secondary skin infections in cancer patients are suspected, it is recommended to perform bacterial and fungal cultures to determine the source of infection before using antibiotic treatment (23).	5	A
Reducing skin irritation	10. Medical staff should guide cancer patients taking EGFRIs to minimize skin irritation (27). Cancer patients should avoid overcleaning their skin as much as possible and refrain from using soaps, detergents, or cosmetics containing ethanol and soap-based ingredients (16, 17).	2	A
	11. Cancer patients should avoid using over-the-counter acne-treatment drugs or topical preparations and other skin-irritating substances to reduce chemical irritation (16, 17).	3	A
	12. Cancer patients should avoid physical stimuli such as rubbing, scratching for itching, hair removal, and shaving (15).	5	A
	13. Cancer patients should minimize going out in cold, dry, or high temperature weather (13).	5	A
Promoting skin comfort	14. Cancer patients should wear loose, soft cotton clothes and comfortable shoes and socks (13).	2	A
	15. Cancer patients should avoid showering with water that is too cold or too hot. It is recommended to bathe with lukewarm water (17).	5	A
	16. Cancer patients can try rinsing the itchy area with lukewarm water and gently patting it dry, or relieve the itching by diverting their attention, such as listening to music or reading (15).	5	B
Skin sun protection care	17. Cancer patients are encouraged to stay away from sunlight and reduce long-term outdoor activities during the afternoon when the sun is shining brightly (29).	2	B
	18. Cancer patients are recommended to use physical sun protection methods such as wearing sun-protective clothing and hats (27).	2	B
	19. Cancer patients are advised to use a broad-spectrum ultraviolet A (UVA)/ultraviolet B (UVB) sunscreen with a sun protection factor (SPF) of 30 or higher daily (12). It is recommended to apply or reapply sunscreen to body parts exposed to sunlight within 1 h before going out and every 2 h outdoors (11, 17).	2	B
Skin moisturizing care	20. Apply a gentle, moisturizing, hypoallergenic emollient that does not contain ethanol but may contain ceramides or other physiological lipids to the skin twice daily (12, 17).	2	A
	21. Cancer patients are advised to apply aloe vera gel topically twice daily to promote skin moisturization (13).	2	B
	22. For areas with relatively high oil secretion, such as the face, the front of the neck, and the back, it is recommended to choose a lightweight lotion. For areas prone to dryness, like the extensor sides of the limbs and the hands and feet, it is advisable to select a thick-textured moisturizing cream or ointment (29).	2	A
Drug preventive measures	23. Within the first 6 weeks of EGFRIs treatment, medical staff can consider using drugs such as antibiotics and low-potency corticosteroids for prevention based on an analysis of the advantages and disadvantages and in combination with the patient’s personal wishes (11, 14, 16, 29).	5	B
Drug treatment measures	24. For cancer patients with grade 1–2 cutaneous toxicity symptoms, the EGFRIs treatment should be continued. For those with grade 3 or higher cutaneous toxicity symptoms, the EGFRIs dosage should be discontinued or adjusted (19, 28, 29).	1	A
	25. Administer antibiotics, corticosteroids, and anti-histamine drugs to cancer patients as per the doctor’s orders to treat cutaneous toxicity symptoms (11, 12, 17).	1	A
	26. In traditional Chinese medicine treatment, the cutaneous toxicity caused by EGFRIs should be treated through syndrome differentiation and treatment from four aspects: the appearance of skin lesions, the nature of deficiency or excess, symptoms, and the location of the affected Zang-fu organs (28). Oral formulas such as Xiaozhen Fang, Modified Yinqiao San, Jiawei Xiaofeng San, and Puxing Jiedu Tang can reduce the incidence and severity of ECFRIs-related rashes and improve the treatment effectiveness (13, 20).	2	B
	27. Cancer patients can use topical preparations such as Honeysuckle Liquid, Zhiyang Pingfu Ye, antibacterial moisturizers, drying gels, preservatives, and hydrocolloid dressings to reduce the severity of cutaneous toxicity caused by EGFRIs and improve the treatment effectiveness (18, 20, 26).	2	B

## 4 Discussion

### 4.1 Scientific validity of evidence

This study conducted a comprehensive literature review on clinical decisions, guidelines, evidence summaries, recommended practices, and expert consensus involving the management of cutaneous toxicity symptoms caused by EGFRIs by systematically searching databases and relevant websites, rigorously screening and conducting quality evaluations of the literature, and extracting and integrating the best evidence. Among the seven included guidelines (13–19), all the evaluation items of AGREE II in three guidelines (13, 16, 19) were “yes,” with a recommended grade of A. The remaining four guidelines (14, 15, 17, 18) had a recommended grade of B, indicating that the formulation process of the included guidelines was relatively rigorous, the methodologies were reliable, and the overall quality was high. The three included evidence summaries (20–22) and two recommended practices (23, 24), as traced through the references, were found to have relatively good overall quality. All the items in the five included expert consensus (25–29) were “yes,” and they were all high-quality documents. Two researchers strictly adhered to the principles of rigor, transparency, science, and standardization. During the processes of evidence screening, extraction, translation, and synthesis, they tried their best to avoid the influence of subjective consciousness and presented the current status of the best evidence in this field with high quality.

### 4.2 Clinical utility of evidence

#### 4.2.1 Professional medical training

Currently, both domestic and international guidelines and expert consensus regarding skin reactions induced by targeted drugs are available. However, the understanding of guideline content and recommended measures among clinical healthcare providers is far from satisfactory (23). Clinical managers should attach great importance to the knowledge needs of healthcare providers regarding targeted drugs, promptly conduct training to fill knowledge gaps, enabling them to master the symptom manifestations, predilection sites, development process, severity grading, treatment principles, and precautions of commonly used drugs for cutaneous toxicity caused by EGFRIs. This way, they can provide higher-quality healthcare services to cancer patients (12, 18, 23, 27). Acne-like rashes represent the earliest and most prevalent toxicity symptoms of EGFRIs (10). Approximately 60% of patients with rashes experience symptoms such as skin pruritus, xerosis, desquamation, a burning sensation, and pain (11), which frequently impact the daily existence and nocturnal slumber of cancer patients (25). Rashes are prone to occur in regions abundant in sebaceous glands (such as the head and face, particularly the nose, cheeks, nasolabial folds, and perioral areas, the “V”-shaped zone of the neck, the upper chest, and the back, etc.). When severe, the limbs can also be affected, and in extreme cases, the entire body can be involved (11, 25). Moreover, rashes caused by EGFRIs generally adhere to a well-defined clinical course. Rashes typically emerge within 1–2 weeks of treatment, peak at 4–6 weeks of treatment, and gradually wane 3–4 months subsequent to treatment (10, 16). The advancement of rashes typically traverses four distinct phases: during the first week, there is paresthesia (dysesthesia) accompanied by erythema and edema; from the first to the third week, red papules and pustules gradually materialize on the skin; from the third to the fourth week, purulent substances can surface on the skin and commence drying to form crusts; during the fourth week, there may be generalized erythema accompanied by scattered telangiectasia (12). Clinically, the diagnosis of cutaneous toxicity symptoms is typically based on a clear record of EGFRIs drug ingestion and pertinent skin clinical symptoms. Unless the clinical manifestations are atypical or the rashes are unresponsive to corresponding treatment, skin biopsy is generally not carried out to confirm cutaneous toxicity symptoms (11, 28). It is recommended that oncologists and dermatologists establish a collaborative team paradigm and enhance the referral and consultation regime. For patients whose cutaneous toxicity symptoms remain unimproved within 2 weeks of continuous treatment, those with moderate-to-severe cutaneous toxicity, and those with an atypical rash appearance or distribution, expert consultation is advisable. It is recommended to assemble a professional cutaneous toxicity team comprising oncology experts, dermatologists, pharmacy specialists, nursing teams, etc. (14, 16). Based on the type, severity, and location of cutaneous toxicity induced by EGFRIs, and in conjunction with the patient’s personal volition, individualized treatment can be administered to each cancer patient to assist them in effectively managing cutaneous toxicity symptoms (24, 25).

#### 4.2.2 Patient health education

Peeters et al. (30) conducted a review of a panitumumab trial involving 463 cancer patients, thereby confirming that the clinical grading of cutaneous toxicity symptoms in cancer patients is associated with patient-reported health outcomes, quality of life, and survival. Acne-like rashes do not represent a contraindication to the continued administration of EGFRIs. A consistent positive correlation exists between the severity of rashes and anti-tumor activity (10, 22), and rashes may serve as a surrogate marker for the therapeutic efficacy of EGFRIs (12, 26). Cancer patients undergoing targeted therapy, often afflicted with malignant tumors and concurrently experiencing drug-related adverse reactions, are confronted with cutaneous toxicity symptoms. In particular, the dense rashes on the face significantly affect their self-image. The pruritic symptoms are generally pronounced, which frequently leads to patient irritability and excessive concern regarding treatment prognosis (31). This situation can potentially disrupt patients’ intimate and interpersonal relationships, even culminating in communication barriers and social isolation. In fact, rashes are non-infectious (28), self-limiting, and reversible (26). They tend to exhibit a pattern of repeated recurrence and remission throughout the treatment cycle, with their severity generally gradually diminishing (29). Typically, they subside within 4 weeks subsequent to the discontinuation of EGFRIs, without leaving any scars (11, 25). It is recommended that medical personnel apprise cancer patients and their families of the clinical manifestations, potential implications, treatment efficacy, and prognosis of cutaneous toxicity symptoms induced by EGFRIs (20, 23). Clearly communicate to cancer patients that EGFRI-induced cutaneous toxicity symptoms can be effectively managed at all possible stages and across all grades, thereby alleviating their psychological burden and enhancing their confidence in treatment (26). Lei and Chen (32) posited that following the administration of drugs to targeted patients, due to the absence of effective nursing management, patients lack an accurate understanding of the adverse reactions ensuing from drug intake and the corresponding countermeasures, resulting in poor medication compliance. This finding underscores the imperative for medical staff to prioritize the health education requirements of cancer patients in relation to targeted therapy. It is recommended that medical staff furnish relevant health education materials and information resources. During hospitalization, through means such as dedicated lectures on cutaneous toxicity symptoms, patient-experience exchange sessions, and the dissemination of educational brochures, and subsequent to patient discharge, via continuous nursing interventions such as telephone follow-up or WeChat-based follow-up, to facilitate cancer patients in enhancing their knowledge of EGFRIs and their self-skin-care capabilities (20, 22).

#### 4.2.3 Precise skin assessment

The cutaneous toxicity symptoms in cancer patients resulting from EGFRIs are associated with both drug-related factors (such as the type of EGFRIs, dosage, treatment duration, etc.) and patient-specific factors (including gender, smoking status, skin phototype, immune status, and history of exposure to cytotoxic drugs, etc.) (10, 12, 29). At present, EGFRIs are principally categorized into two main classes: tyrosine kinase inhibitors (TKIs) and monoclonal antibodies (mAbs) (33). Research has uncovered that the cutaneous toxicity reaction spectra of EGFR-TKIs and EGFR-mAbs bear resemblance; nevertheless, the occurrence rate of cutaneous toxicity symptoms is marginally higher in the case of EGFR-mAbs compared to EGFR-TKIs, with minute disparities in incidence among various drugs (34). These cutaneous toxicity symptoms commonly exhibit dose-dependence and frequently follow a pattern of recurring and remitting cycles over the course of treatment (10, 29). A retrospective research endeavor demonstrated that male cancer patients are more disposed to develop moderate-to-severe rashes, often resulting in the discontinuation of EGFRIs treatment, in contrast to their female counterparts (35). The correlation between age and the risk of cutaneous toxicity remains an enigma. One study indicated an association between patients aged 70 years or older and an elevated risk of severe rashes in non-small-cell lung cancer patients (36). In contrast, another study proposed a link between patients aged 70 years or younger and a higher incidence of grade 3 rashes in metastatic colon cancer patients (37). Currently, the evidence supporting the impact of age on cutaneous toxicity symptoms is scarce, and cancer patients of any age group are susceptible to these symptoms (10, 11). Smoking has the potential to stimulate the production of pigments within liver cells (11), which in turn accelerates the clearance rate of EGFRIs drugs (12), consequently increasing the maximum tolerated dose of EGFRIs in cancer patients. As a result, smokers typically experience less severe cutaneous toxicity symptoms compared to non-smokers (28). A study has revealed that moderate-to-severe rashes are almost solely observed in cancer patients with a skin phototype I or II (38), suggesting that the skin phototype represents a potential determinant influencing the cutaneous toxicity symptoms in cancer patients (11). Additionally, elements such as the immune status of cancer patients and the occurrence of genetic mutations like Kirsten rat sarcoma (KRAS) mutations can also impact their cutaneous toxicity manifestations (28). The utilization of cytotoxic drugs and concurrent radiotherapy in cancer patients is prone to impair the skin’s barrier function (20). Exposure of the skin to ultraviolet rays and inadequate moisturization might play a supplementary role in the progression and severity of rashes, thereby exacerbating the cutaneous toxicity symptoms of patients (11).

Assessment represents the initial step in symptom management. Healthcare providers should make a clinical diagnosis of cutaneous toxicity symptoms based on the EGFRIs medication history of cancer patients and the results of physical examinations (12). It is recommended that healthcare providers evaluate aspects such as the location, nature, severity, density, development process, triggering factors, presence of infection, patients’ subjective experiences, and reports and records of other adverse reactions of the cutaneous toxicity symptoms caused by EGFRIs (17, 20). Precise assessment of cutaneous toxicity symptoms is conducive to scientifically assisting clinical decision-making and formulating accurate treatment strategies. Currently, common skin assessment tools include CTCAE, MESTT, EGFRISTI, DLQI, Skindex, FACT-EGFRIs-18, etc. (14, 18, 20, 26). The Common Terminology Criteria for Adverse Events (CTCAE) is the most prevalent skin evaluation tool in current clinical trials and management reviews. It simply categorizes the cutaneous toxicity symptoms of EGFRIs based on the affected body surface area and symptoms (11). However, its limitations lie in its inability to reflect the sub-acute or chronic nature of cutaneous toxicity symptoms, and the patient-reported outcomes are not incorporated into the assessment of subjective symptoms and the impact on quality of life (12). Clinical healthcare providers should fully consider the objective circumstances of clinical practice when using this tool, select appropriate assessment items, and avoid omissions that could lead to incomplete reports (11). The MASCC EGFRI Skin Toxicity Tool-MESTT (39) was developed by the Multinational Association of Supportive Care in Cancer (MASCC). It has a strong correlation with the grading of CTCAE and can address the under-reporting and insufficient assessment of skin-related adverse events (18). The Epidermal Growth Factor Receptor Inhibitor Related Skin Toxicity Index (EGFRISTI) (40) determines the severity of cutaneous toxicity through numerical quantification. This tool is recommended for assessing the scope of cutaneous toxicity of EGFRIs (12). Furthermore, the Dermatology Life Quality Index (DLQI) (41), Skindex series scales (42, 43), and the Functional Assessment of Cancer Therapy-Epidermal Growth Factor Receptor Inhibitor-18 (FACT-EGFRIs-18) scale (44) are all patient-reported tools and belong to skin-related quality-of-life scales. They are recommended for use in the quality-of-life assessment of cancer patients after they have used EGFRIs. It is recommended that clinical healthcare providers assess cancer patients at least once every 2 weeks to promptly identify and intervene in cutaneous toxicity symptoms (26). If secondary skin infection in cancer patients is suspected, it is advisable to conduct bacterial and fungal cultures before using antibiotic treatment to determine the source of infection (23).

#### 4.2.4 Reducing irritation and promoting comfort

EGFR plays a crucial role in regulating skin inflammation, barrier function, and innate immunity (45). Affected by EGFRi drugs, the skin of cancer patients is more vulnerable compared to normal skin. Therefore, medical staff should guide them to minimize skin irritation (27). First, cancer patients should avoid over-cleaning their skin and avoid showering with water that is too cold or too hot. It is recommended to use lukewarm water at ≤40 °C (17). Second, cancer patients should avoid using skin-irritating substances such as soaps, detergents, or cosmetics containing ethanol and soap-based ingredients; over-the-counter acne-treatment drugs or topical preparations, etc. (16, 17). Ingredients such as ethanol and soap-based substances easily absorb the moisture in the skin, exacerbating the cutaneous toxicity symptoms of cancer patients (21). The rashes caused by EGFRIs are called acne-like rashes because their papules, nodules, and pustule-like skin lesions resemble acne, but they do not have the typical comedone characteristics of acne (12). If cancer patients use over-the-counter acne-treatment drugs or topical preparations such as *α*-hydroxy acids and benzoyl peroxide gel on their own, this may irritate and worsen the acne-like rashes, exacerbate skin dryness, and increase itching (26). Cancer patients should reduce going out in cold, dry, or hot weather; wear loose, soft cotton clothing and comfortable shoes and socks; and avoid sun exposure (13). Try to reduce the patient’s desire to scratch for itching as much as possible. Avoid excessive rubbing and scratching to prevent skin damage and subsequent infection. They can try rinsing the itchy area with lukewarm water and gently patting it dry, or relieve the itching by diverting their attention, such as listening to music or reading (15). Cancer patients should try to avoid waxing and plucking. Male patients should avoid using razors to shave. They can use electric shavers or non-abrasive shaving methods, such as simply trimming beards and hair with scissors, to prevent skin scratches (21).

#### 4.2.5 Sun protection and moisturization care

Ultraviolet exposure can inhibit EGFR expression in skin keratinocytes and is one of the risk factors for cutaneous toxicity symptoms caused by EGFRIs, facilitating the occurrence or exacerbation of rashes (11). Medical staff should instruct cancer patients to take good personal sun protection measures, encourage them to stay away from sunlight, and reduce long-term outdoor activities from 10:00 to 15:00 h (29). Cancer patients can use physical sun protection methods such as wearing sun-protective clothing, hats, and using umbrellas (27), or use sunscreen to reduce potential skin damage caused by ultraviolet exposure. It is recommended that cancer patients use a broad-spectrum UVA/UVB sunscreen with a sun protection factor (SPF) of 30 or higher daily (12), and apply or reapply it to body parts exposed to sunlight within 1 h before going out and every 2 h outdoors (11, 17). In addition, cancer patients should regularly use emollient lotions or creams to effectively moisturize their skin and support post-sun repair. It is recommended to apply a gentle, moisturizing, hypoallergenic emollient that does not contain ethanol (12, 17). Medical skin care products containing ceramides or other physiological lipids and having skin–barrier–repair effects are preferred. For areas with relatively high oil secretion, such as the face, front of the neck, and back, cancer patients can choose a lightweight lotion and apply it twice daily. For areas prone to dryness, such as the extensor sides of the limbs, hands, and feet, cancer patients can choose a thick-textured moisturizing cream or ointment and apply it twice daily (29). Aloe vera gel has the effects of activating the vitality of skin cells, effectively locking in skin moisture, and nourishing the skin. Cancer patients can consider applying aloe vera gel topically twice daily to promote skin hydration (13).

#### 4.2.6 Drug preventive measures

Some experts have pointed out that compared with reactive treatment of cutaneous toxicity symptoms using antibiotics, prophylactic antibiotic treatment is linked to a reduced risk of rashes across all grades. Thus, a preemptive prevention strategy is anticipated to enhance the quality of life of cancer patients (12). It is recommended that medical staff, after analyzing the pros and cons and considering the patient’s personal wishes, think about using medications like antibiotics and low-potency corticosteroids for prevention in the first 6 weeks of EGFRIs treatment (11, 14, 16, 29). Tetracycline-class antibiotics such as doxycycline, minocycline, or oxytetracycline can be chosen and taken twice daily for a total of 6 weeks. In cases of intolerance or a relevant allergy history, alternative antibiotics include cephalosporin-class antibiotics such as cefadroxil, trimethoprim-sulfamethoxazole, all taken twice daily (11, 17, 22). Cancer patients can also opt to apply low-potency topical corticosteroids like hydrocortisone and alclometasone, either alone or in combination, twice daily to skin areas such as the face and chest (11, 17). This approach is especially suitable for cancer patients with a history of skin diseases such as psoriasis, eczema, and atopic dermatitis, and for high-risk groups whose skin tends to be dry and itchy (29).

#### 4.2.7 Drug treatment measures

For cancer patients with grades 1 and 2 cutaneous toxicity symptoms, the EGFRIs treatment should be continued. For those with grade 3 or higher cutaneous toxicity symptoms, the EGFRIs dosage should be discontinued or adjusted (19, 28, 29). In addition, cancer patients should use antibiotics, corticosteroids, and antihistamine drugs as per the doctor’s orders to treat cutaneous toxicity symptoms (11, 12, 17). Common topical corticosteroids include hydrocortisone, alclometasone, mometasone valerate, or fluocinonide cream, etc. Common topical antibiotics include clindamycin, dapsone gel, etc. It is recommended that all of the above-mentioned drugs be used twice daily for at least 2 weeks (11, 12). If medical staff reevaluate after 2 weeks and find that the symptoms have not improved or have worsened, oral tetracycline-class antibiotics such as doxycycline or minocycline can be used in combination, twice daily for 4–6 weeks. Systemic glucocorticoids can also be used for a short term to treat cutaneous toxicity symptoms (11, 12). Medical staff should suspect secondary viral or bacterial infections based on the morphology of skin lesions (such as yellow scabs or secretions, redness around the lesions) and the degree of purulent exudate. Before using antibiotics, the exudate should be cultured to determine an appropriate antimicrobial treatment plan (11, 23). In cases where cancer patients are intolerant or have a relevant history of allergies, alternative antibiotics include cephalexin, cefadroxil, trimethoprim-sulfamethoxazole, etc., all of which are taken twice daily (11, 17, 22). When cancer patients feel unbearably itchy, they can choose topical antipruritics such as menthol, pramoxine, doxepin cream, or take oral anti-histamine drugs to relieve the itching (17, 27). For daytime itching, non-sedating second-generation anti-histamine drugs such as loratadine are the first choice; for patients with unbearable itching at night that affects sleep, first-generation sedating anti-histamine drugs such as diphenhydramine and hydroxyzine can be considered. It is worth noting that antihistamine drugs should be used with caution in elderly cancer patients. Before use, medical staff must fully consider the interactions between drugs, especially when used in combination with central nervous system inhibitors (12). Generally, skin dryness symptoms can be effectively alleviated through daily moisturizing measures. However, when it is severe enough to cause cracking, in addition to daily moisturizing measures, drugs containing urea and glycerin can be used to promote skin smoothness. Cyanoacrylate preparations, salicylic acid ointment, or propylene glycol solution can be used for soaking to relieve skin pain and promote healing (18, 27).

In traditional Chinese medicine, the etiology and pathogenesis of EGFRI-induced cutaneous toxicity are mainly due to the cancer patients’ own insufficient constitution, making them vulnerable to pathogenic toxins. Additionally, they are affected by the special toxicity of EGFRI drugs. External pathogens invade the interstitial spaces of the skin. Unable to be drained internally or penetrated externally, they stagnate and transform into heat, consuming blood and injuring yin. Deficiency of blood generates wind and dryness, leaving the skin lacking nourishment (28). According to the different stages of pathogenic factors, traditional Chinese medicine treatment often focuses on dispersing the lung qi, clearing heat, cooling the blood, and removing blood stasis to relieve the excess symptoms. Patients with rashes are classified into four syndromes: wind-heat in the lung meridian, damp-heat in the stomach and intestines, yin deficiency with internal heat, and stasis-heat and phlegm accumulation (46). Various empirical oral formulas such as Xiaozhen Fang (47), Modified Yinqiao San (48), Jiawei Xiaofeng San (49), and Puxing Jiedu Tang (50) have been developed, achieving good curative effects in cancer patients. These formulas can reduce the incidence and severity of ECFRI-related rashes, improve the treatment effectiveness, and enhance the quality of life of patients (13, 20). Honeysuckle has the effect of clearing heat and detoxifying. Researchers soaked sterile gauze (4–6 layers) with Honeysuckle Liquid until it was just about to drip. Then, they applied it closely to the affected skin. The frequency of wet-dressing depends on the severity of the rash, generally 3–6 times daily, 20–30 min each time, with a suitable temperature of 38–40 °C. After continuous treatment for 1 week, it was found that the cutaneous toxicity symptoms of cancer patients using cetuximab were effectively relieved (51). A multi-center randomized controlled trial showed that Zhiyang Pingfu Ye has good antibacterial and anti-inflammatory effects. It can effectively reduce the severity of cutaneous toxicity symptoms caused by EGFRIs in cancer patients, with high patient satisfaction and clinical application value (52). In addition, topical preparations such as antibacterial moisturizers, drying gels, preservatives, and hydrocolloid dressings can also reduce the severity of cutaneous toxicity caused by EGFRIs (18, 20, 26).

## 5 Conclusion

This study summarized the best evidence for managing cutaneous toxicity symptoms in cancer patients caused by EGFRIs, including 27 pieces of evidence across 9 aspects: professional medical training, patient health education, precise skin assessment, reducing skin irritation, promoting skin comfort, skin sun protection care, skin moisturizing care, drug preventive measures, and drug treatment measures. These can serve as a reference for clinical prevention and treatment of cutaneous toxicity symptoms caused by EGFRIs. Most of the recommended opinions in this study are of Grade A, indicating that the results of this study are relatively reliable. Our research group has published an independent parallel sub-study focusing specifically on EGFRI-induced paronychia (53). The two studies adopted independent literature retrieval schemes, extracted distinct evidence items and generated separate clinical practice recommendations. Due to overlapping submission, revision and proofreading schedules of the two papers, the cross-citation was accidentally omitted during proof correction, with no intention to conceal related published work. Readers may refer to both articles to obtain a comprehensive overview of our team’s full evidence-based research system covering multiple categories of EGFRI-associated adverse events. Our findings indicate that the clinical feasibility and generalizability of the preventive use of the antibiotic strategy still have deficiencies. It is hoped that more large-sample, multi-center, high-quality literature will be carried out in the future to develop a unified theoretical system and practical operation norms to provide more scientific and standardized guidance. This study has limitations in its literature search, confined to Chinese and English databases, potentially introducing language bias and missing relevant evidence. It should be noted that while relevant literatures on Traditional Chinese Medicine (TCM) were incorporated during the literature retrieval process, this review did not systematically cover the potential contributions of other traditional medicine systems, such as Indian Ayurveda. Future research could further explore the role of these traditional medical approaches in preventing and managing adverse reactions induced by EGFRIs, with the aim of providing more comprehensive supportive care options for patients. Additionally, it must be acknowledged that data extraction and synthesis, despite adhering to standardized procedures, inherently involve an element of subjective interpretation, which poses a risk of interpretation bias. Healthcare providers need to comprehensively evaluate the feasibility of measures for managing cutaneous toxicity symptoms caused by EGFRIs in the actual clinical application context, fully consider the patient’s wishes, follow the principle of individualization, and prudently apply the evidence to clinical practice. At the same time, healthcare providers should thoroughly analyze the obstacle factors and facilitating factors of evidence application, formulate targeted action strategies, implement changes at the individual and organizational levels, and embed high-quality evidence into clinical practice. The management strategies for cutaneous toxicities summarized in this study are primarily symptom-based, serving as a universal foundation in current clinical practice. However, future research should further investigate potential disparities in skin toxicity profiles across different cancers, which may arise from variations in the types of EGFR inhibitors, combination regimens, and patient baseline characteristics. Although existing evidence supports a generalized management approach, recognizing these differences is critical for advancing toward precision prevention. Well-designed literature is imperative to explore cancer-specific risk features, thereby paving the way for a transition from universal supportive care to individualized, preemptive management strategies.

## Data Availability

The raw data supporting the conclusions of this article will be made available by the authors, without undue reservation.
