# Correction: Best evidence summary for the management of epidermal growth factor receptor inhibitor-induced skin toxicity symptoms in cancer patients

**DOI:** 10.3389/fmed.2026.1927907

**Published:** 2026-10-05

**Authors:** Kexuan Li, Xiuyue Qiu, Jiayu Zhang, Yingshuyi Zhou, Youfen Fan, Qionghui Sun

**Affiliations:** 1 Burn Department, Ningbo No.2 Hospital, Ningbo, Zhejiang, China; 2 School of Nursing, Zhejiang Chinese Medical University, Hangzhou, Zhejiang, China; 3 School of Nursing, Zhejiang Changzheng Vocational & Technical College, Hangzhou, Zhejiang, China; 4 Department of Gastrointestinal Surgery, Ningbo No.2 Hospital, Ningbo, Zhejiang, China

**Keywords:** cancer, targeted therapy, epidermal growth factor receptor inhibitors, skin nursing, summary of evidence

Reference “53. Li K, Qiu X, Zhang J, Zhou Y and Fan Y (2025) Best evidence summary for preventing and preventing epidermal growth factor receptor inhibitors induced paronychia in cancer patients. Front. Oncol. 15:1555114. doi: 10.3389/fonc.2025.1555114” was erroneously omitted.

The parallel study focusing on EGFRI-induced paronychia (53) was not cited in the article. The citation has now been inserted in the Conclusion section and should read:

“This study summarized the best evidence for managing cutaneous toxicity symptoms in cancer patients caused by EGFRIs, including 27 pieces of evidence across 9 aspects: professional medical training, patient health education, precise skin assessment, reducing skin irritation, promoting skin comfort, skin sun protection care, skin moisturizing care, drug preventive measures, and drug treatment measures. These can serve as a reference for clinical prevention and treatment of cutaneous toxicity symptoms caused by EGFRIs. Most of the recommended opinions in this study are of Grade A, indicating that the results of this study are relatively reliable. Our research group has published an independent parallel sub-study focusing specifically on EGFRI-induced paronychia (53). The two studies adopted independent literature retrieval schemes, extracted distinct evidence items and generated separate clinical practice recommendations. Due to overlapping submission, revision and proofreading schedules of the two papers, the cross-citation was accidentally omitted during proof correction, with no intention to conceal related published work. Readers may refer to both articles to obtain a comprehensive overview of our team's full evidence-based research system covering multiple categories of EGFRI-associated adverse events. Our findings indicate that the clinical feasibility and generalizability of the preventive use of the antibiotic strategy still have deficiencies. It is hoped that more large-sample, multi-center, high-quality literature will be carried out in the future to develop a unified theoretical system and practical operation norms to provide more scientific and standardized guidance.”

The original version of this article has been updated.

